# Modelling the persistence of mosquito vectors of malaria in Burkina Faso

**DOI:** 10.1186/s12936-018-2288-3

**Published:** 2018-04-02

**Authors:** Ace R. North, H. Charles J. Godfray

**Affiliations:** Department of Zoology, University of Oxford, Woodstock Road, Oxford, OX2 6GG UK

**Keywords:** *Anopheles gambiae*, *Anopheles coluzzii*, Mosquito aestivation, Mosquito migration, Model

## Abstract

**Background:**

Populations of the *Anopheles gambiae* complex are found during the rainy season throughout West Africa, even in arid areas with long dry seasons during which mosquitoes appear to be absent. Several hypotheses have been proposed to explain this apparent paradox, including aestivation, dispersal between neighbouring settlements, and long distance migration using high-altitude wind currents.

**Methods:**

An individual-based, spatially explicit model of mosquito populations was developed for a region of West Africa centred on, and including all of, Burkina Faso. Populations associated with human settlements were linked by dispersal and the model incorporated geospatial data on the distribution of settlements, water bodies and rainfall.

**Results:**

Local dispersal (at rates consistent with experimental data) was necessary to explain observed patterns of rainy season populations across all of the simulation area, but by itself failed to account for the presence of populations in the arid North (the Sahel). The presence of rare dry-season larval sites could explain these northern populations, but seems inconsistent with field surveys. Aestivation by female mosquitoes explained rainy-season populations in all but the very sparsest and driest areas of human habitation, while long-distance migration based on annual wind patterns could account for all observed populations.

**Conclusions:**

Modelling studies such as this can help assess the potential validity of different hypotheses and suggest priority areas for experimental study. In particular, the results highlight a shortage of empirical research on mosquito dispersal between neighbouring settlements, which may be critically important to the continued presence of many mosquito populations in West Africa. Further research that establishes the extent to which mosquitoes aestivate, and migrate using high altitude winds, is also much needed to understand Sahelian mosquito populations.

**Electronic supplementary material:**

The online version of this article (10.1186/s12936-018-2288-3) contains supplementary material, which is available to authorized users.

## Background

Despite enormous progress in recent years, malaria remains hyper-endemic in large parts of sub-Saharan Africa [1]. The most severely afflicted region is West Africa which contains eight of the ten countries that had the highest incidence in 2015 (Mali, Burkina Faso, Guinea, Côte d’Ivoire, Togo, Nigeria, Sierra Leone, and Benin; [1]). To understand why malaria is so persistent here and to refine control methods, it is critical to understand the population biology of malaria vectors in the region.

The four major mosquito vectors of malaria in Africa—*Anopheles gambiae* sensu stricto (s.s.), *Anopheles coluzzii, Anopheles arabiensis,* and *Anopheles funestus*—are all present in sub-Saharan West Africa [2], and all undergo population dynamics driven by a highly seasonal cycle of rainfall (until molecular diagnostics were developed the first three species were lumped in the taxon *Anopheles gambiae* sensu lato (s.l.), but the narrow concept of the species is used below). There is a rainy season, typically between July and November, with the rest of the year being hot and dry. The duration and amount of rain during the rainy season varies markedly along a North–South axis (the northernmost part, the West African Sahel, being the driest) and also from year to year in any given location [3]. Mosquito populations increase dramatically each rainy season due to the creation of larval sites in puddles and along the edge of temporary streams, and then decline when these sites dry up as the rainy season ends. The precise timing of population growth and decline differs amongst vector species [4–8], for reasons that are not fully resolved though a key factor is thought to be how each species survives the dry season [8]. In much of the region, mosquito populations seem to disappear completely in the dry season only to reappear at the onset of the rainy season [4, 5, 8, 9], even in very arid regions. How mosquito populations are maintained during the dry period remains unclear, despite decades of research, though there are three main hypotheses [4].

First, it has long been known that that some adult mosquitoes hide in (largely unknown) shelters during the dry season in a semi-dormant state (aestivation; [9, 10]). There is mounting evidence that this behaviour plays a role in the persistence of some *An. coluzzii* populations [8, 11–15], though there remains less evidence that other anophelines aestivate [8]. Second, localities might be colonised at the start of the rainy season by mosquitoes dispersing from nearby locations where there are persistent populations owing to year-round surface water. Although there is good evidence that this occurs in some locations ([4, 16] (West Africa); [17, 18] (East Africa)), it seems an unlikely explanation for vector persistence in parts of the Sahel which are remote from permanent water (for example at least 30 km; [13, 14]). The third possibility is that mosquitoes migrate long distances by passive transport in high-altitude winds to recolonise habitats each rainy season. This hypothesis dates back to the 1960s [19], yet it has received less attention in recent decades which may reflect the difficulty of obtaining evidence of mosquitoes travelling large distances. However, interest has revived following the publication of trap data from a Sahelian village in Mali, where *An. gambiae* were undetected in the dry season yet abundant in the latter part of the rainy season [8]. The nearest large (and year-round) populations of *An. gambiae* are at least ~ 150 km from this study site, hence migration spanning hundreds of kilometres appears necessary to explain the observed dynamics [8].

It is of course possible that these processes combine to allow vector persistence throughout the region, in a manner that depends on location as well as species. In the case of *An. coluzzii*, recent genetic data from a Malian Sahel village (the same study site as [8]) has suggested that this population survives the dry season predominantly by aestivation (in agreement with [8]), yet long distance migrants also arrive over the course of a rainy season [15]. However, two studies comparing populations of *An. coluzzii* from locations close to or more distant (at least 30 km) from permanent surface water, found traits indicative of aestivation only in the remote populations (reduced oviposition despite having access to water [13]; reduced flight activity [14]).

This paper develops a simulation model of the dynamics of a mosquito species to explore vector persistence in Burkina Faso and the surrounding region. The model is intended to represent either *An. gambiae* or *An. coluzzii*, which are probably the most efficient malaria vectors in the world [20, 21]. The distributions of these sibling species differ in West Africa, though they are sympatric in much of the study area [2, 22]. Although the two species have somewhat different ecologies [22–25], the model is used to investigate entomological parameters that are uncertain for both species, in particular those relating to dry season ecology and movement behaviour.

The model is individual-based [26] and assumes that the structure of the mosquito metapopulation in the study region can be represented by the ensemble of connected populations associated with individual human settlements. This assumption is made because *An. gambiae* and *An. coluzzii* are well known to be highly anthropogenic [21, 27–30]. The individual-based nature of the current model allows stochasticity to be considered in a natural manner, which means that population sites may become extinct when conditions are unfavourable and later recolonised if conditions become favourable. The model incorporates geospatial data on human settlements, water courses (rivers and lakes), and rainfall.

Several other large-scale models of mosquito dynamics in Africa have been developed that adopt different assumptions about mosquito population dynamics. For example the Liverpool malaria model (LMM) [31–33] and the Open Malaria Warning (OMaWa) model [34, 35] are deterministic which improves computational efficiency though makes it harder to represent stochasticity. A number of stochastic and spatially explicit models have been described [36–38], though these were intended for the investigation of anopheline dynamics across small areas (village-sized [36, 37]) or medium areas (region-sized, ~ 1000 km^2^ [38]), and their simulation on a country-sized area would be computationally prohibitive. The large and medium scale models all represent African mosquito metapopulations as the ensemble of populations at the points of a lattice. This again is computationally efficient though makes it harder to incorporate the geographical heterogeneity that may affect population persistence, and also makes it more difficult to compare model results with field surveys that are typically based at human settlements. The approach used here would be less suitable to mosquito species with low rates of anthropophily, or which breed in areas with sparse human habitation, where a lattice formulation may be preferable. Compared to LMM and OMaWa, a relatively simple model for mosquito dynamics is used, that assumes population growth rates are not influenced by temperature and humidity. This is done in order to focus on the role of variation in rainfall and groundwater (and for computational efficiency) and this assumption is returned to in the discussion.

The aim of this work was to explore how assumptions about mosquito behaviour affect population persistence across an area of West Africa that is large enough to exhibit the wide variation in environmental conditions found in this region. In particular, the model is used to address the observation that mosquito populations occur during the rainy season in the driest locations in the study area. The results suggest that population persistence in these areas requires either aestivation, long-distance movements or the presence of year-round breeding sites, and the different effects these processes have on mosquito population dynamics are explored.

## Methods

An individual-based model was developed to simulate mosquito populations in an area of West Africa centred on, and including, Burkina Faso. The model takes account of spatial variation in rainfall patterns, the presence of rivers and lakes, and the distribution of humans. The mosquito population across the entire simulation area is treated as a network of local populations located at human settlements, and it is assumed that local populations are connected to neighbouring populations through the short-distance dispersal of adult mosquitoes. For some simulations, it is assumed in addition that more distant populations are connected by the long distance migration of adult females. The maximum density of a local population is assumed to be influenced by both the amount of rainfall at a given time, and the presence and extent of local water bodies (which may be seasonal or permanent).

### Simulation region

**Fig. 1:**
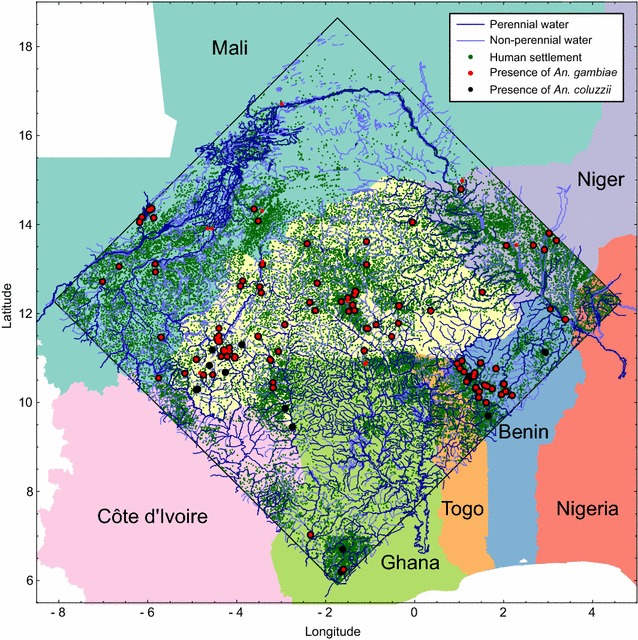
The simulation area, delineated by the square whose sides are 1000 km^2^. Note that the black and red presence markers show all the locations where entomological studies have been performed (there are no absence markers; [2])

The model is set it a region of West Africa with sides of 10^3^ km centred on the mid-point of Burkina Faso (longitude − 1.737°, latitude 12.274°). This region includes all of Burkina Faso and territory of seven neighbouring countries (Mali, Niger, Nigeria, Benin, Togo, Ghana, and Côte d’Ivoire; Fig. 1). A recent review [2] collated studies that recorded the presence of malaria vector species in different locations across Africa, and this included 330 records of *An. coluzzii* and 321 records of *An. gambiae* in 150 sites within the study region (Fig. 1).

### Distribution of human settlements

The model uses settlement data collected by the United Nations Office for the Coordination of Humanitarian Affairs (OCHA), which is available from the Humanitarian Data Exchange (HDX; https://data.humdata.org/). These data give the location, name, region and country of villages, towns, and cities throughout the region (larger towns and cities may also be subdivided into smaller settlement units), comprising 42,360 human settlements in total (Fig. 1).

### Distribution of water bodies

Inland water data extracted from the digital chart of the world (DCW), which includes all significant water courses in the region (available from http://www.diva-gis.org/Data), is used to set the distribution of water bodies. Rivers and other water bodies are represented as a series of segments (lines or polygons), each of which is classified as either “non-perennial, intermittent, or fluctuating” or “perennial or permanent”. The edges of the polygon segments (representing lakes and wide rivers) were extracted from these data, and treated as linear habitat elements since *An gambiae* s.l. tends to breed at the periphery of large water bodies [25]. In total, these data comprise 63,299 km and 61,953 km of non-perennial and perennial water courses within the simulation region (Fig. 1).

### Rainfall

Rainfall data were obtained from the “ERA-interim reanalysis”, covering the period January 1st 1979 to February 28th 2017 at a spatial resolution of 0.5 degrees (longitude and latitude), which is available from the European Centre for Medium-Range Weather Forecasts [39]. The data were aggregated to obtain weekly total rainfall for each grid square. There is considerable variation in the amount of rain falling across the simulation region, though most parts experience a rainy season between August and September and are driest between December and April (see Additional file 1).

### Mosquito demographic model

Each human settlement is assumed to represent the potential site of a mosquito population which is made up of juveniles, adult males, virgin females, and mated females. The juvenile category includes all immature mosquito stages from egg to pre-reproductive adult, which is assumed to last for *T*_*L*_ days (treated as a constant). The simulation model keeps track of the numbers of individuals in each life-stage, which are updated each day using pseudo-random draws from appropriate probability distributions.

Each day, juveniles either die or age by 1 day. The probability that a juvenile survives is $p_{s} = \sqrt[{T_{L} }]{{\frac{{\alpha \left( {x,t} \right)}}{{\alpha \left( {x,t} \right) + J_{T} }}}}\left( {1 - \mu_{J} } \right)$ where $\mu_{J}$ represents density independent mortality, $J_{T}$ is the total number of juveniles in the population, and $\alpha \left( {x,t} \right)$ is a variable controlling the strength of mortality due to larval competition for resources ($\alpha \left( {x,t} \right)$ is the number of juveniles at which the probability of death from larval competition over the course of development is 0.5). This quantity varies over space (*x*) and time (*t*) because it is influenced by rainfall and the presence of local water bodies (see below).

Adults die with probability $\mu_{A}$ per day and over the same period virgin females mate with probability $p_{m} = \frac{M}{\beta + M}$, where $M$ is the number of males in the population and $\beta$ is the adult male population size at which the daily probability of mating is 0.5. This parameter is set at the low value of 100, so that females almost always mate on their first day after emergence in all but very small populations. This is on the assumption that there are very few old virgin females in anopheline populations. Unfortunately, no data was found that would allow this parameter to be estimated more accurately.

Each day a mated female lays a number of eggs, which is Poisson distributed with expectation $\theta$. All juveniles when they reach age $T_{L}$ emerge to become an adult male or virgin female with equal probability.

Adults disperse locally with probability $d$ to sites within a neighbourhood of radius of $L_{D}$. Within this area, the probability of moving from focal site *i* to site *j* (from within the *k* sites in the neighbourhood) depends on their distance apart, $d_{ij}$, and is $\frac{{L_{D} - d_{ij} }}{{\mathop \sum \nolimits_{k} (L_{D} - d_{ik} )}}$. Note that the distribution of distances for local dispersal events depends on the location of the focal settlement—dispersal from settlements with many neighbours is typically more short-range than dispersal from isolated settlements. This contrasts with ‘lattice’ spatial models, where populations are typically equidistant (e.g. [34, 38]). An additional figure plots the variation in the mean distance of a dispersal event across all settlements in the simulation area (see Additional file 2).

### Influence of water courses and rainwater

The presence of local water bodies influences larval competition and thus carrying capacity. It is assumed that a population at location $x$ at time $t$ has a value of the function, $\alpha (x,t)$ that determines local maximum density,

1 $$\alpha \left( {x,t} \right) = \alpha_{0} (x) + \alpha_{1} \left( {1 - e^{{ - \phi r\left( {x,t} \right)}} } \right) + \alpha_{2} \left( {1 - e^{{ - \kappa \left[ {W_{p} \left( x \right) + W_{n} (x)\left( {1 - e^{{ - \delta r\left( {x,t} \right)}} } \right)} \right]}} } \right).$$

The first term, $\alpha_{0} (x),$ is assumed absent in the baseline version of the model and is returned to below.

The second term represents the contribution to breeding habitats from rainfall $r(x,t)$ through, for example, the creation of puddles and other small water bodies in which *An. gambiae* s.l. is known to breed. In the absence of rainfall this term is zero, but as precipitation rises it increases at a rate determined by the parameter $\phi$ to asymptote at a level $\alpha_{1}$. Recall that rainfall is averaged by week so that, at any given location $x$, $\alpha (x,t)$ varies on a weekly rather than daily basis.

The final term represents the larval sites associated with rivers and lakes which may be permanent, $W_{p} (x)$, or intermittent, $W_{n} (x)$. The magnitude of the two components is calculated from the length of water courses of the two types within a radius of $L_{w}$ of the focal population at $x$. It is assumed that intermittent water courses are replenished by rainfall at a rate determined by the parameter $\delta$, providing the same density of breeding habitat as permanent sites when rainfall is heavy. Mosquito carrying capacity increases at a rate determined by the parameter $\kappa$ as the density of both types of water body grows, the influence reaching an asymptote at $\alpha_{2}$.

### Mosquito aestivation

Aestivation is modelled by supposing that there is a period in each year $(t_{A1} ,\;t_{A2} )$ when mated females enter a dormant state with daily probability $\psi$, which they survive with probability $1 - \mu_{E}$. If a female does survive, she emerges on a random day within a later period $(t_{A3} ,\;t_{A4} )$, and resumes her normal activities.

### Long-distance migration

Long distance migration is modelled by supposing that there is a period each year $(t_{D1} ,\;t_{D2} )$ when mated females initiate migration from the north-east to the south-west with probability $d_{M}$ each day, and a second period $(t_{D3} ,\;t_{D4} )$ when migration is from the south-west to the north east (migration is assumed to initiate with the same probability in each direction). The timing and direction of long-distance movement were chosen to reflect the annual wind patterns in this region [3]. A female survives migration with probability $1 - \mu_{M}$, in which case the female moves to a new site chosen at random from a sub-region of the simulation area that extends from the focal site in a NE $\to$ SW direction (or vice versa) to the edge of the simulation area. The distribution of mean migration distances (in either direction) obtained from a model simulation with default parameters is plotted in Additional file 2.

### Small permanent larval sites

It is possible that there is a class of permanent breeding habitats that is always present, irrespective of rainfall, but are not of the size that are present in the permanent water body database (hereafter ‘small permanent larval sites’). It is unclear whether this type of larval site is important to mosquito biology and in the basic version of the model they are assumed to be absent. The term $\alpha_{0} \left( x \right)$ in Eq. (1) is used to explore their effect on mosquito population persistence. It is assumed that small permanent sites are lognormally distributed across local populations with mean $\alpha_{0}^{\mu }$ and variance $\alpha_{0}^{{\sigma^{2} }}$.

### Model parameters

All the model parameters and their default values are shown in Additional file 3. The demographic life-history parameters $(T_{L} , \;\mu_{J} ,\;\mu_{A} ,\;\theta )$ are taken from the literature [40–43]; Lambert et al. pers. comm.). The carrying capacity parameters $(\alpha_{0} ,\;\alpha_{1} ,\;\alpha_{2} ,\;\phi ,\;\kappa ,\;\delta )$ were determined using mosquito population size estimates from three villages in the region (two in Burkina Faso and one in Mali) and through expert judgement. Further details and justification of the default parameters are provided in Additional file 4.

## Results

### Local dispersal

**Fig. 2:**
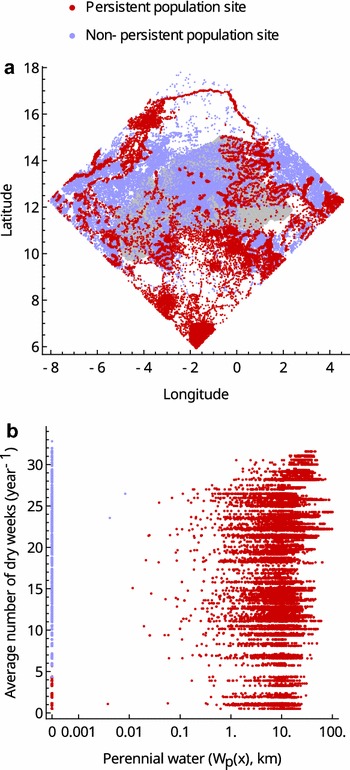
The distribution (**a**) and characteristics (**b**) of settlements that, in the simulations, persist indefinitely in isolation (without immigration or aestivation; red points). Settlements where populations become extinct in these conditions are shown as blue dots

Consider first a version of the model where aestivation and long-distance migration do not occur, and there are no residual breeding habitats ($\psi = d_{LDM} = \alpha_{0} (x) = 0$). If the dispersal of mosquitoes between neighbouring settlements is also disallowed, so that the model represents a set of isolated populations, a majority (~ 56%) of settlements are without a local mosquito population after 10 years. Mosquitoes persist in the remaining settlements because they are adjacent to permanent water bodies, or because they are in locations where rainfall occurs throughout the year (in the southernmost part of the region), or both (Fig. 2; these settlements will henceforth be referred to as harbouring ‘persistent populations’). Recall that field surveys find nearly all settlements have viable mosquito populations in the rainy season. The model thus suggests that local dispersal may be important to explain this observation.

**Fig. 3:**
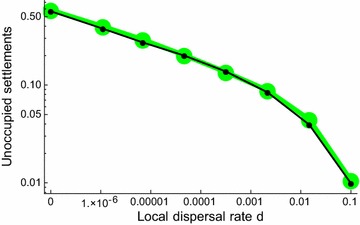
The effect of local dispersal on the fraction of settlements that do not maintain a population during the wet season. The black line shows the results of the standard model for which adult mortality is constant irrespective of adult age, while the green line shows results of the same model except for an assumption that adults die at age 30 days if not before

Allowing local dispersal amongst settlements does result in more mosquito populations during the rainy season (Fig. 3). There is some information on local dispersal rates in West Africa from mark-release–recapture experiments in The Gambia [44], Burkina Faso [45], and Mali [46]. In each study, a number of *An. gambiae* s.l. marked with fluorescent powder were released in one village with traps being placed in both the focal and neighbouring villages. From these experiments lifetime probabilities of movement between villages of between 0.04 and 0.24 were estimated. Assuming the default daily mortality rate of 0.125 these estimates translate to a rate of dispersal (the parameter *d*) of 0.005–0.034.

With local dispersal rates of 0.005 the percentage of settlements without mosquitoes in the rainy season falls from ~ 56 to ~ 5%, and with dispersal rates of 0.034 to ~ 2%. However, to replicate observed patterns of mosquito populations (less than 1% settlements without mosquitoes in the rainy season) much higher local dispersal rates are required, approximately 10% of mosquitoes moving amongst settlements per day (*d* = 0.1), which seems unrealistically high.

**Fig. 4:**
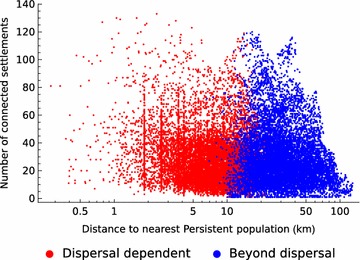
The effect of proximity to a persistent population and the local density of settlements on settlement status. With dispersal *d *= 10^−5^ (and aestivation, migration, and small permanent larval habitat all turned off), the model predicts that 25% of settlements are regularly colonised in the wet season (red), and 31% are not (blue). The x-axis plots distance to the nearest of the remaining 44% of sites that are permanently occupied (even with no dispersal), and the y-axis plots the total number of sites within 10 km from the focal site

Local dispersal might increase the numbers of settlements occupied during the rainy season by allowing persistent populations to act as sources of mosquitoes that colonise less favourable locations each season (sometimes called island-mainland metapopulation dynamics), or the dispersal may allow the existence of a “blinking-light” or classical metapopulation where non-persistent populations often persist for several generations, sending out their own colonists, before going extinct. Both processes may occur simultaneously but examination of the model results suggests that the former is most important. The probability that a site is occupied during the rainy season is more correlated with its distance to the nearest persistent population than to the local density of settlements (Fig. 4). In a classical metapopulation, higher settlement density tends to be correlated with occupation rates because of a network effect where nearby populations are able to rescue each other from extinction.

The basic implementation of the model assumes a constant mortality hazard (and hence an exponential survival curve) which means that when populations are high a small fraction of individuals survive for a long time, possibly through the dry season. The model was rerun assuming a maximum insect longevity of 30 days because of a concern that these very old mosquitoes, an artefact of the survival function assumption, might affect the mosquito distribution. This change had a negligible effect on population incidence (Fig. 3).

**Fig. 5:**
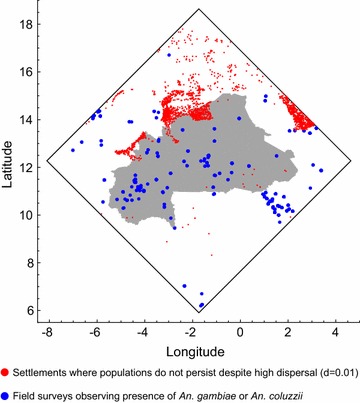
Red points mark settlements where the simulations predict populations cannot persist despite frequent local dispersal (*d *= 0.01), if there is no aestivation, migration, or unknown permanent breeding habitat. Blue points mark the locations of field studies, of which all have identified the presence of *An. gambiae* or *An. coluzzii*

If the local dispersal parameter, *d*, is 0.01, which is in the middle of the range of the mark-recapture estimates described above, the number of settlements that remain unoccupied in the rainy season each year varies between 1500 and 2000 (~ 4% of all settlements). These are mainly confined to the northern part of the simulation region (Fig. 5). Though there have been fewer surveys of mosquitoes in the north compared to the south of the region, those that have been carried out have found viable populations of *An. gambiae* s.l. in all settlements that were sampled (Fig. 5). The model is now used to explore the three non-exclusive hypotheses to explain the persistence of these populations: aestivation, long distance migration, and residual permanent breeding habitat.

### Aestivation

**Fig. 6:**
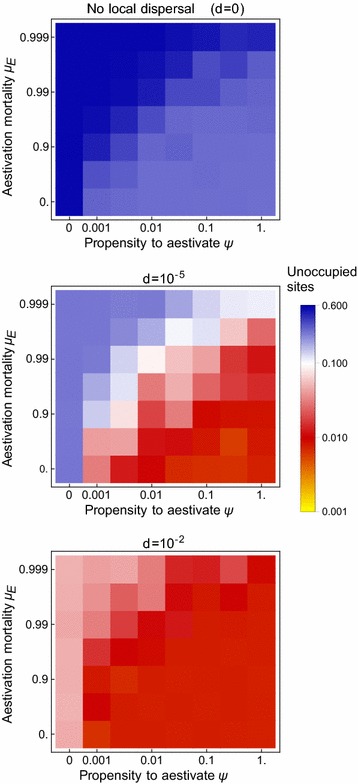
The effect of aestivation on population incidence during the late rainy season (on the 19th October), if aestivation and local dispersal are the only processes supporting dry season persistence

The effect of aestivation on the proportion of settlements with rainy season mosquito populations is shown in Fig. 6 for three different assumptions about the extent of local dispersal between settlements. An increasing propensity to aestivate, and higher survival over the dry season, both lead to more rainy season mosquito populations. For example, when one in 100 mosquitoes enter aestivation per day, and 10% survive the dry season, the percentage of settlements without rainy season mosquitoes drops to around 1%, as long as there is some local dispersal. However, further aestivation does little to reduce this percentage and the number of unoccupied sites does not drop below ~ 300 for any of the parameter combinations shown in Fig. 6.

**Fig. 7:**
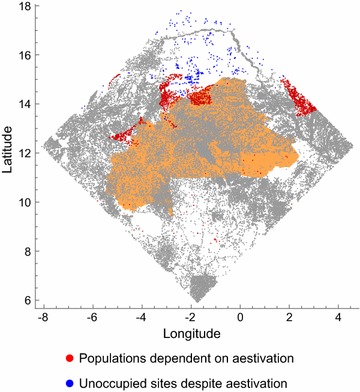
The distribution of populations that are dependent on aestivation (red) and sites that are unoccupied despite aestivation (blue), when local dispersal rate d = 0.01. Aestivation is assumed to occur with propensity $\psi = 0.01$ and mortality $\mu_{E} = 0.9$. Gray dots are sites that are occupied even in the absence of aestivation

Aestivation has the greatest effect on mosquito population dynamics in more arid areas. Figure 7 plots those settlements which, if moderate amounts of dispersal are assumed, only have rainy season populations in the presence of mosquito aestivation. The populations are concentrated in an arid area of the Burkina Faso-Mali border, and further east at the edge of the study region in Niger. Also shown in the figure, in blue, are the remaining 300 settlements with no mosquito populations. These are chiefly in central Mali, an area that is both the driest in the region (and without permanent water bodies) and also with the sparsest pattern of human settlement (Fig. 1).

### Migration

**Fig. 8:**
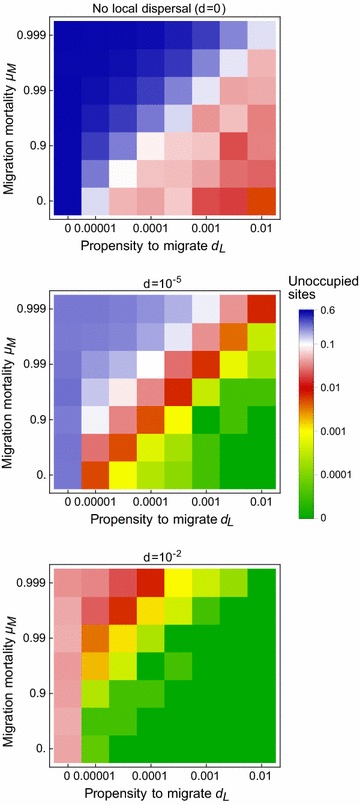
The effect of migration on (rainy season) population incidence if migration and local dispersal are the only processes supporting local persistence (cf. Fig. 6)

**Fig. 9:**
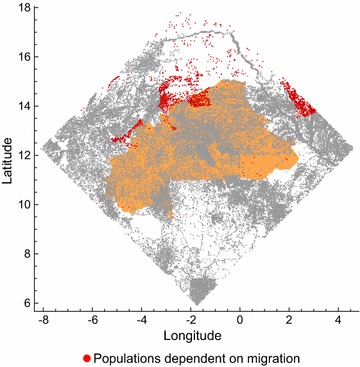
The distribution of populations that are dependent on migration (red), when local dispersal rate d = 0.01. Migration is assumed to occur with propensity $d_{L} = 0.001$ and mortality $\mu_{M} = 0.99$. Gray dots in are sites that are occupied even in the absence of migration

The fraction of settlements in the region without mosquito populations during the rainy season declines if long-distance migration is permitted (Fig. 8). If the propensity to migrate and the probability of surviving migration are high enough, every settlement is colonised during the rainy season, even those in the most arid part of the north of the study region (Fig. 9).

### Small permanent larval sites

**Fig. 10:**
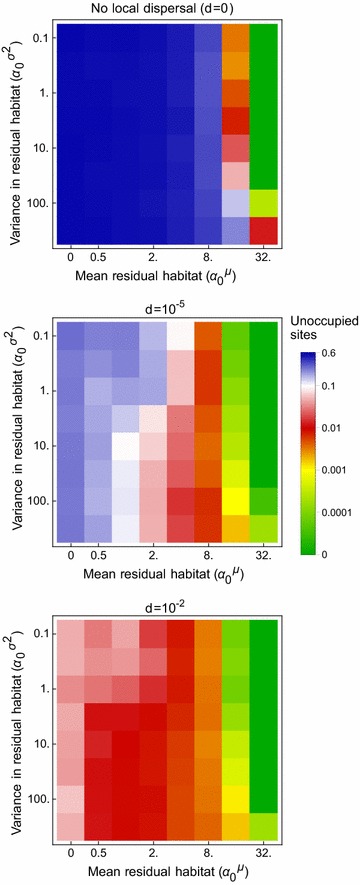
The effect of assuming there are small permanent larval sites (and neither aestivation nor migration) on (rainy season) population incidence

Unsurprisingly, increasing the average amount of assumed permanent habitat leads to more settlements having mosquitoes during the rainy season (Fig. 10). If enough of this habitat exists, widespread rainy season populations can be explained. Increasing variance tends to reduce rainy season incidence though in the absence of local dispersal, both the mean and variance have to be high to make a major difference. When mosquitoes are allowed to disperse locally, and more settlements are occupied during the rainy season, increasing variance can mitigate some of the effects of lower mean levels of small permanent breeding habitat. This appears to occur because some habitations have large mosquito populations that are able to colonise other settlements in the vicinity.

### Effects of dry season ecology on local population dynamics

Periodic surveys of mosquito abundance allow local population fluctuations to be observed in the field, and have been used to investigate how specific populations might be maintained [8, 12]. The model is now used to examine how the different dry season hypotheses influence local population dynamics.

**Fig. 11:**
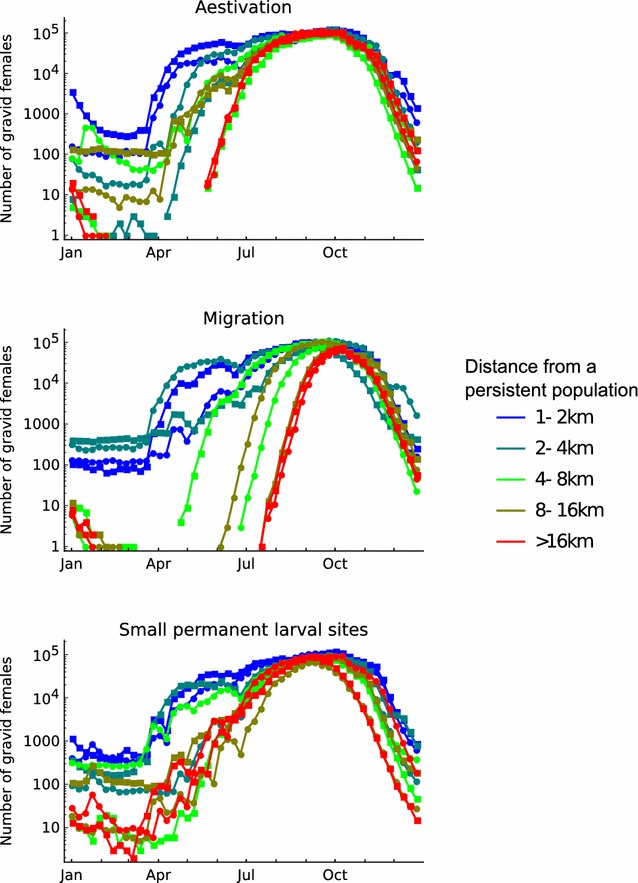
Local dynamics at random selected sites depending on dry season ecology and the distance of the site to a persistent population. Aestivation parameters are $\psi = 10^{ - 3} ,\mu_{E} = 0.9$, migration parameters are $d_{L} = 10^{ - 5} ,\mu_{M} = 0.99$, and small permanent larval site parameters are $\alpha_{0}^{\mu } = 4,\alpha_{0}^{{\sigma^{2} }} = 10$

For a given parameterisation, simulations predict that there is substantial variation among sites in the timing of population growth at the start of the rainy season, and in the numbers of mosquitoes present in the dry season (if there are any). Despite this variation, there are consistent patterns in the population dynamics depending on whether persistence is maintained by aestivation, migration, or by small permanent larval sites (shown in Fig. 11). If aestivation is important, the number of active females increases rapidly at the start of the rainy season at approximately the same time across sites. If migration is more important, the population growth phase tends to be later (by up to a month), though there is considerable variation in timing across sites, reflecting the highly stochastic nature of colonisation by females migrating long distances. If a population persists because of the presence of permanent larval habitat, active adult mosquitoes will be present throughout the dry season albeit at very low numbers. In this case population growth occurs early but slowly at the very beginning of rainy season, so that there is a less distinct spurt after the first major rain. These differences are most evident in sites that are distant from persistent populations, where the effects of local dispersal per se are less important.

## Discussion

This study explores the factors allowing the continued presence of mosquito populations in West African locations where larval habitat is scarce for many months each year. Computer simulations cannot of course prove which processes are involved but are important for exploring hypotheses and guiding empirical work.

The simulation results suggest that local dispersal amongst settlements is important in maintaining mosquito populations in the rainy season right across the study region. Extinction during the dry season occurs relatively frequently and without local dispersal there are no insects present to exploit breeding opportunities at the start of the rainy season. There have been some estimates of local dispersal rates from a limited number of mark-release–recapture experiments [44–46] and these seem to be high enough to explain why mosquitoes are present during the rainy season across most of the study area. However, further experimental and possible genetic data would be valuable to confirm this conclusion.

Even if local movements are very frequent, the model predicts that there are settlements in the Sahelian part of the study area which should not have viable mosquito populations in the rainy season. Though there have been fewer field surveys in the arid north of the study region compared to the south, those that have been conducted regularly record mosquitoes in the rainy season [2] (and similarly in other parts of the Sahel outside the simulation area (e.g. [8, 14])). This suggests that there is more to population persistence than local dispersal alone, a conclusion also reached by entomologists after studying mosquito autecology [8]. Small permanent larval sites, absent from the water-course data base used to build the model, might have a widespread presence during the dry season. There are many types of largely man-made larval sites that fall in this category including wells and other water containers, and small reservoirs which can be found on perennial streams in Burkina Faso [47]. In East Africa, *An. gambiae* s.l. larvae have been found in a wide range of man-made water bodies, including sewage ponds and drains in the urban environment of Dar es Salaam [48], artificial containers and holes on a rural island in Lake Victoria [49], and cement-lined pits in a rural town next to Lake Victoria [50]. Unfortunately, no general surveys of the distribution and significance of small man-made larval sites in West Africa were available. Even if quite rare, the model found they can explain the maintenance of viable populations during the dry season. However, several field studies investigating the dry season ecology of *An. gambiae* s.l. conducted extensive surveys for potential larval sites without finding them (e.g. [11, 12]). Though some cryptic larval sites will affect mosquito population dynamics, it appears unlikely that their existence is by itself a complete explanation for all extant rainy-season populations. A number of previous large-scale models of mosquito dynamics in Africa have assumed there is a low background rate of recruitment into the adult population during the dry season (presumably from larvae in rare small water bodies) [31–33].

Evidence of *An. gambiae* s.l. aestivation dates back to the late 1960s [9, 10] and more recently a number of studies have reported this behaviour in some *An. coluzzii* populations [8, 11–15]. However, except for one mark-release–recapture experiment that caught a single female mosquito that had been marked in the previous rainy season (some 7 months earlier, [11]), the evidence is indirect, being based on measurements of phenotypic traits [9, 10, 13, 14], time-series of trap data [8, 12], or genetic analyses [15].

Incorporating aestivation into the model led to rainy-season populations in most locations, but not in the most northern (Sahelian) locations which have the lowest rainfall and the longest dry seasons. This is, in part, because populations do not become large in these locations due to the low rainfall even in the rainy season. However, it is possible that the current model understates the potential for population growth during the rainy season in the Sahel, where mosquito predation is generally lower than in locations with persistent mosquito populations (T. Lehmann, pers. comm.). Furthermore, mosquitoes are thought to use rainfall cues, in particular to time their emergence from aestivation after the first big rain of the year (T. Lehmann, pers. comm.), a strategy that is likely to be selected in a region such as the Sahel with considerable year to year variation in when the rainy season starts [3]. An interesting extension of this work will be to investigate whether more detailed models of aestivation, that incorporate these factors, would affect the conclusions. Unfortunately, at present too little is known about aestivation behaviour to explore such behaviours. Other large-scale models have assumed that a small number of adult mosquitoes enter an age class (more than 44 days old) with relatively low mortality and which allows some to survive the dry season by, in effect, aestivating [34, 35]. They too find aestivation can help explain rainy-season mosquito populations.

Evidence for long-distance migration of *An. gambia*e s.l., like that for aestivation, is indirect and based on survey data [8] and genetic [15] analyses. The available information is thus limited and long-distance migration was incorporated in this model in a deliberately simple way based on broad seasonal patterns of air flow that can be revised as more is discovered. However, the simulations suggest that relatively low levels of long distance movement by mated females could give rise to most settlements being occupied during the rainy season. Further study of long-distance movement would be very informative.

It is not necessary for mosquitoes to aestivate in every settlement, or for each to be recolonised by long-distance migrants, to obtain widespread rainy season populations. As long as there is some local dispersal, the model simulations showed that populations that successfully survive the dry season through aestivation or are recolonised by long-distance migrants can seed other populations in their vicinity where neither occurred. It may be possible to detect this effect by looking at the detailed timing of rainy-season resurgence as a function of local settlement density and connectedness, though such data is not currently available. The three hypotheses explored in this study give rise to different patterns of local population dynamics, particularly in those settlements that are some distance from persistent populations (Fig. 11). In these settlements there may be substantial variability in dynamics between years, regardless of the dry season ecology, so that inferences of the dry season ecology from survey data alone will require good quality data spanning multiple years. However, such data are relatively easy to collect by field entomologists, and this approach has been used to investigate dry season anopheline behaviour in a Sahelian village in Mali [8]. Analysis of the time-series reported in this study and comparison with the model outputs are most indicative of aestivation in the case of *An. coluzzii* and migration in the case of *An. gambiae* and *An. arabiensis* [8]. Of course, the three hypotheses are not mutually exclusive. In the study village used by [8], more recent genetic evidence has suggested that *An. coluzzii* populations both aestivate and are reinforced by migrants [15]. Simulations including multiple factors in general found their effects combined additively except that aestivation made little further difference in the presence of rare dry-season larval sites (see Additional file 5).

The model presented in this paper is particularly motivated by the biology of *An. gambiae* and its closely related and morphologically indistinguishable sister species *An. coluzzii*, both of which show strong anthropophily [21, 27–30]. This is the basis of the assumption that the spatial distribution of mosquito populations corresponds to the pattern of human settlement. There is a further morphological indistinguishable species of the *An. gambiae* complex, *An. arabiensis*, which is also a significant vector of malaria in the region. This species varies in its degree of anthropophily [21] though appears to be more associated with humans in the West African part of its range [51]. It is found in drier environments compared with *An. gambiae* and *An. coluzzii,* though even less is known about its aestivation and migration behaviour. There are further subtle differences in the ecology of these three closely related species [22–25] that would be interesting to include in future versions of the model. For instance it is well established that *An. coluzzii* more than *An. gambiae* is able to exploit large irrigated fields, in particular rice paddies, as breeding sites [6]. However, based on current understanding of the ecology of the three species it has not been possible to develop species-specific versions of the model hence the decision to work with a generic *Anopheles* model.

As mentioned in the introduction, the current model deliberately omits some aspects of mosquito biology to reduce the times it takes to run the simulation and to allow us to concentrate on broad spatial patterns. In particular, it is assumed that temperature and humidity do not directly influence mosquito survival and development (though rainfall of course is a major driver of habitat availability). The modelling approach can easily be extended to allow weather-dependent mosquito demographic parameters. It would also be possible to include a more sophisticated representation of rainfall. At present it is assumed that the number of larval sites increases with rainfall though it is known that heavy rain can, in some environments, be detrimental to mosquitoes as it flushes out their larval larval sites [52]. Further developments could include modelling the movement of water downstream and the creation of larval larval sites as streams turn into pools when rainfall or water flow decreases [53]. It would also be possible to study multi-annual climate variation, for instance consecutive years with above or below average rainfall. Analysis of 200 years of historical precipitation data has shown that there is significant autocorrelation in annual rainfall in this region [3]. The challenge is not so much building the model (though these refinements come at computational expense) as estimating its parameters. Models of this type may be helpful in exploring the timing of specific control measures.

Relatively little is known about local and long-distance dispersal, density dependent processes, adult longevity and aestivation in the *An. gambiae* complex. New information on all these aspects would greatly improve the ability to model mosquito spatial population dynamics.

One type of data that has become much more readily available recently is high resolution geographical data, such as the human settlement, water course and rainfall information that was incorporated in the model. It is likely that the (OCHA) settlement data used here does not include all human settlements in the region. Comparison with satellite data from the Center for International Earth Science Information Network (CIESIN; http://www.ciesin.org/data/hrsl/) suggests that the OCHA data includes all large villages and towns where the two data coincide (CIESIN data is currently available for Burkina Faso, Ghana, and Côte d’Ivoire, though coverage is not complete), but not all small settlements. This suggests that the current model may underestimate connectivity between settlements and, therefore, may underestimate the persistence of mosquito populations. The quality of geographical data—from satellite imagery and other methods—is likely to improve; for example, databases on human settlement will increasingly include richer information about their size and economic development, and possibly even directly relevant variables such as bed net use.

Distinguishing between these hypotheses is important in improving current and developing new methods of mosquito control. For example, were aestivation to be the predominant mode of dry season survival then it might be possible to target vulnerable mosquitoes in their aestivation sites [11]. If long-distance migration is important than novel control proposals such as the use of gene drive introduced into source populations may prove highly effective. In general the individual-based nature of the current model makes it straightforward to adapt to study novel methods of vector control such as gene drive.

## Conclusions

The modelling described here has allowed investigation of the common explanations for the persistence of mosquito populations in West Africa. In much of the study area, the analysis suggests that persistence is the result of mosquitoes dispersing locally from source populations, where there is year-round breeding habitat, into settlements that otherwise would not support populations. However, the extent of this explanation depends critically on the frequency with which mosquitoes move amongst neighbouring populations, of which there remains great uncertainty. Further experimental and genetic studies are much needed.

The model simulations also suggest that some populations are not maintained only by the local dispersal of mosquitoes from populations with year-round breeding habitat, even if local dispersal is very frequent. Both aestivation and long distance migration were investigated, and the model predicted that migration is able to replicate vector persistence in all locations under certain conditions, yet aestivation is not. The failure of aestivation occurred in the driest and remotest locations where populations, if they exist, remain small. Further modelling will help to identify how aestivation behaviour can explain persistence in these locations. These results identify regions where field work to determine dry season persistence may be more efficacious. The model described in this paper can be extended in a number of directions, and thus applied to further issues in vector biology.

## Additional files

**Additional file 1.** The seasonal and spatial trends in rainfall across the simulation area.

**Additional file 2.** Showing the distributions of local dispersal and migration distances across the simulation area.

**Additional file 3.** Model parameters and default values.

**Additional file 4.** Describing how the default parameters were set.

**Additional file 5.** The effects of combining dry season survival hypotheses (aestivation, migration and small permanent larval sites) on vector persistence across the simulation area.
