# Supplementary material for: Modelling the persistence of mosquito vectors of malaria in Burkina Faso
Source: Malar J. 2018 Apr 2;17:140. doi: 10.1186/s12936-018-2288-3 (PMC5879775; doi:10.1186/s12936-018-2288-3)
Supplement: Supplementary file 1 — Additional file 1. The seasonal and spatial trends in rainfall across the simulation area. [file 12936_2018_2288_MOESM1_ESM.pdf]

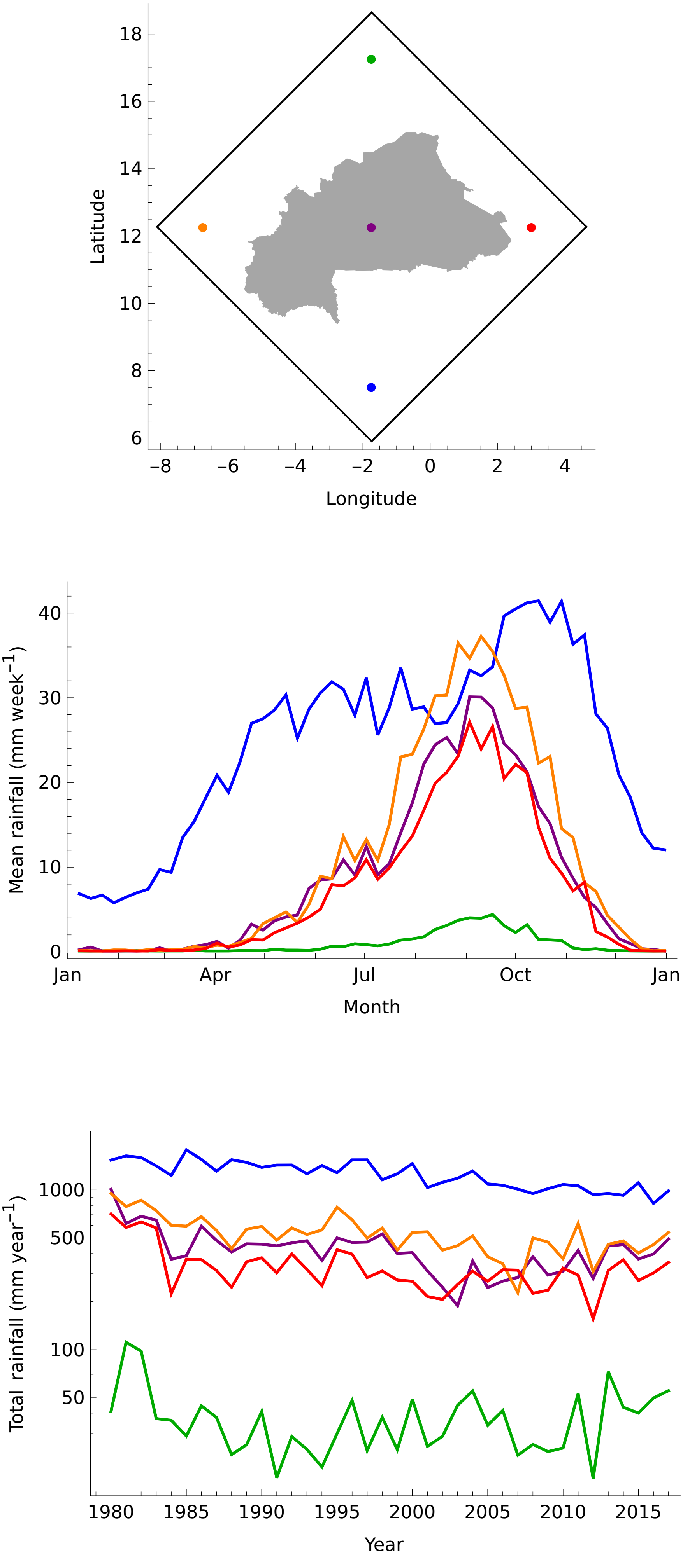

Figure S1: Rainfall across the simulation area. (b) and (c) plot rainfall profiles for five locations across the simulation region marked as points in (a)
