# Supplementary material for: Modelling the persistence of mosquito vectors of malaria in Burkina Faso
Source: Malar J. 2018 Apr 2;17:140. doi: 10.1186/s12936-018-2288-3 (PMC5879775; doi:10.1186/s12936-018-2288-3)
Supplement: Supplementary file 2 — Additional file 2. Showing the distributions of local dispersal and migration distances across the simulation area. [file 12936_2018_2288_MOESM2_ESM.pdf]

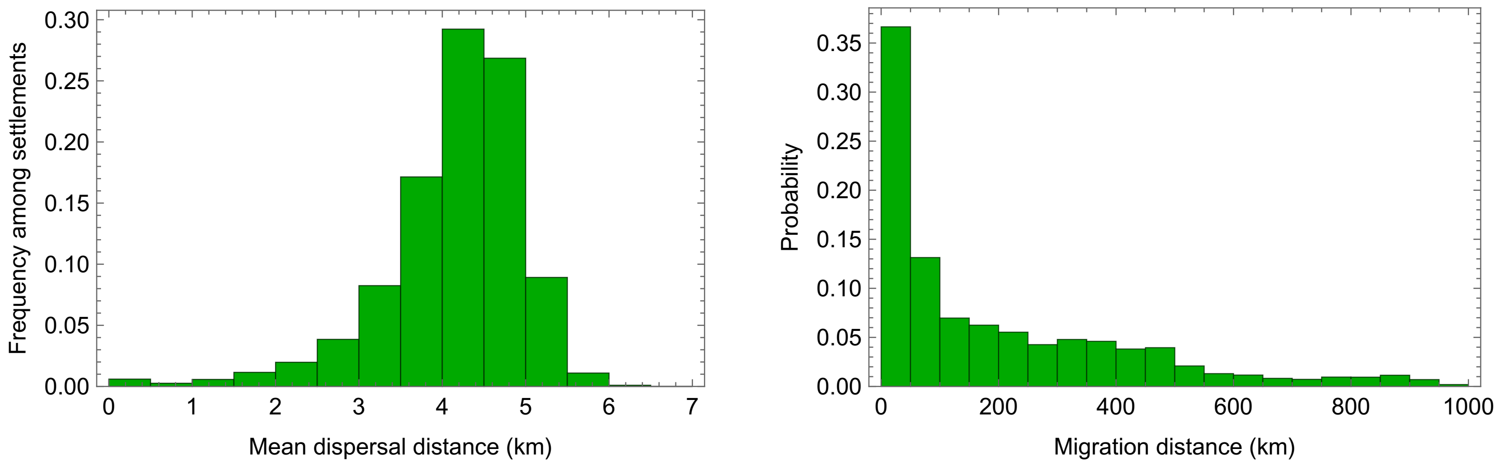

Figure S2: The distributions of local dispersal and migration distances across the simulation area.
