# Supplementary material for: Modelling the persistence of mosquito vectors of malaria in Burkina Faso
Source: Malar J. 2018 Apr 2;17:140. doi: 10.1186/s12936-018-2288-3 (PMC5879775; doi:10.1186/s12936-018-2288-3)
Supplement: Supplementary file 3 — Additional file 3. Model parameters and default values. [file 12936_2018_2288_MOESM3_ESM.pdf]

Parameter	Interpretation	Units	default
	**Demographic parameters**		
T_L	Larval duration	days	10
μ_J	Larval density independent mortality	day^{-1}	0.05
μ_A	Adult mortality	day^{-1}	0.125
θ	Oviposition rate	(mated female)$^{-1}$(day)$^{-1}$	9
β	Density of males when mating rate = 1/2(unmated female)$^{-1}$(day)$^{-1}$	-	100
	**Carrying capacity** $\left[\alpha(x,t) = \alpha_0 + \alpha_1(1 - e^{-\phi r(x,t)}) + \alpha_2(1 - e^{-\kappa[W_p(x)+W_n(x)(1-e^{-\delta r(x,t)})]})\right]$		
α_0	Baseline contribution	-	0
α_1	Maximum contribution from rain *per se*	-	2×10^5
α_2	Maximum contribution from water bodies	-	2×10^5
ϕ	Increase in $\alpha(x,t)/\alpha_1$ per mm rain per week (when rainfall low)	(mm rain)$^{-1}$week^{-1}	0.03
κ	Increase in $\alpha(x,t)/\alpha_2$ per km standing water (within 5km; when water bodies rare)	(km water)$^{-1}$	0.8
δ	Increase in length of standing water from non-permanent waterways per km non-permanent waterways (within 5km) per mm rain per week (when rainfall low)	(mm rain)$^{-1}$week^{-1}	0.03
	**Spatial parameters**		
d	Probability adult disperses to a connected village	day^{-1}	0.001
L_d	Maximum distance at which populations are connected to each other	km	10
L_w	Maximum distance at which populations are connected to water bodies	km	5
	**Aestivation**		
t_{A_1}	Day mated females begin aestivation	date	27th October
t_{A_2}	Day mated females cease aestivation	date	16th December
t_{A_3}	Day mated females begin emerging from aestivation	date	20th May
t_{A_4}	Day mated females finish emerging from aestivation	date	19th June
ψ	Probability female goes into aestivation	day^{-1}	0
μ_E	Probability female dies during aestivation	-	0.95
	**Long distance migration**		
t_{D_1}	Day mated females begin NE→SW migration	date	1st January
t_{D_2}	Day mated females end NE→SW migration	date	30th January
t_{D_3}	Day mated females begin SW→NE migration	date	20th July
t_{D_4}	Day mated females end SW→NE migration	date	19th August
d_M	Probability female migrates	day^{-1}	0
μ_m	Probability female dies during migration	-	0.95

Table S1: Model parameters
