# Supplementary material for: Modelling the persistence of mosquito vectors of malaria in Burkina Faso
Source: Malar J. 2018 Apr 2;17:140. doi: 10.1186/s12936-018-2288-3 (PMC5879775; doi:10.1186/s12936-018-2288-3)
Supplement: Supplementary file 4 — Additional file 4. Describing how the default parameters were set. [file 12936_2018_2288_MOESM4_ESM.pdf]

Additional file 4: Setting model parameters

March 15, 2018

The model parameters are shown in Additional File 3.

1 Demographic parameters

Consider a single isolated wildtype population. To investigate the roles of the demographic parameters we first assume there is no emigration ($d = 0$), no aestivation and no long distance migration. Figure 1 plots the effects of the demographic parameters $\{\mu_J, \mu_A, \alpha_{\text{Max}}, \beta, \theta\}$ on population size (number of adult females).

1.1 Larval (density independent) development

We draw on two studies to select baseline larval development parameters T_L and μ_J, Gimnig et al. (2002) and Muriu et al. (2013), which both explored larval development of *Anopheles gambiae s.l.* in semi-field condition in Kenya.

1.1.1 T_L, larval duration

Across four treatments (two densities and two treatments of larval water - with or without cow dung), Gimnig et al. (2002) measured larval development times between 7.9 and 10.4 days (averages within each treatment). There was a significant effect of larval density (higher density slowed development), but not of other factors. Muriu et al. (2013) measured development times (to pupation, which itself lasts 1-3 days) ranging from 5 days to 20 days across all density treatments. Again there was a significant effect of larval density.

In our model we make the assumption that larval density affects survival but not development time. We set T_L at 10 days which is well within the distributions observed by Gimnig et al. (2002) and Muriu et al. (2013).

1.1.2 μ_J, larval density independent mortality day^{-1}

We can estimate this from measurements of larval survival when larval density is low. Gimnig et al. (2002) estimated larval survival at around 0.6 irrespective of initial density (0.494-0.606 across two densities and two treatments of larval water). Although density did not have a significant effect on survival, it affected both development time and adult mass. Muriu et al. (2013), by contrast, found a significant effect of density on larval survivorship, with probability of pupation in the lowest density treatment ≈ 0.8 when predators were excluded (average across replicates at different times of year) and ≈ 0.6 when predators were not excluded. If we assume there is no mortality from competition in these experiments, we can calculate μ_J as

$$\mu_j = 1 - [\text{Probability of pupation}]^{1/T_L}$$

So if the probability of pupation is 0.6 and $T_L = 10$, we have $\mu_j \approx 0.05$. In fig. 1 we plot population sizes when $\mu_j \in (0.01, 0.1)$.

1.2 Adult (density independent) parameters

1.2.1 μ_A, adult mortality day^{-1}

For *An. gambiae s.l.*, a meta-analysis of MRR data (Lambert Et. Al., unpublished) suggests adult lifespans are very short, on average 2-4 days. A meta-analysis of dissection data (Lambert Et. Al., unpublished) gives longer estimates, in the region of 5-12 days. We suspect the MRR estimate is downwardly biased. As a baseline, we set average adult longevity at 8 days (equal in males and females), giving $\mu_A = 1/\text{longevity} = 0.125$. The effect of variation in this parameter on population size is illustrated in fig. 1.

1.2.2 θ, Oviposition rate day^{-1}

The batch size of a female *An. gambiae s.l.*, defined as the number of eggs she lays in a single gonotrophic cycle, varies by numerous factors including species, body size, environment, and dietary conditions (Gary and Foster, 2001; Yaro et al., 2006; Manda et al., 2007; Yaro et al., 2012). Two studies using field caught An. gambiae s.s. and An. coluzzii have measured batch sizes in the range 150-200 (Yaro et al., 2006), and 100-200 (Yaro et al., 2012), with the variation depending mostly on female body size (Yaro et al., 2006) and time of year (Yaro et al., 2012). Two lab experiments have measured oviposition rates of *An. gambiae s.l.* across a variety of dietary treatments. Gary and Foster (2001) measured eggs per day per female in the range 9-13.0 depending on whether the female is offered sugar as well as blood. Manda et al. (2007) explored the effects of different plant sugars on fecundity and measured batch sizes in the range 15-80 depending on treatment (type of plant sugar) and whether the females were offered one or three blood meals. In this experiment, approximately 60% eggs hatched across treatments.

We convert the results from Yaro et al. (2006), Manda et al. (2007), and Yaro et al. (2012) to estimate the number of viable eggs that escape predation per day per female (our parameter θ) as (observed batch size)×(hatching rate)×(1-egg predation)/(gonotrophic cycle length). We expect significant egg predation in field environments, though of course this will vary considerably. For observed batch size we select 120 eggs which is well within the range across studies, a 60% hatching rate, a 50% predation rate and assume a cycle duration of 4 days, giving eggs per day per female $= \theta = 9$, which is also at the lower end of the range measured by Gary and Foster (2001) (which did not consider predation or hatching rate).

1.3 Density dependent parameters

1.4 β, Density of males when mating rate $= 1/2$(unmated female)$^{-1}$(day)$^{-1}$

We are unaware of data that can be used to estimate the effect of male density on female mating rate in field conditions. It is expected that population growth should be possible from very few adults in favourable conditions, suggesting a low value of β. The size of a population at equilibrium is barely affected by the value of β for all but unrealistically large β (fig. 1). However, the value of β may play a role in determining the recolonisation of empty habitat sites, and the extinction of populations suppressed by a gene-drive.

We set a low default of $\beta = 100$.

1.5 α_{Max}, larval density when mortality from competition $= 0.5$ in optimal conditions

We let α_{Max} take the value of $\alpha_0 + \alpha_1 + \alpha_2$ whose values we discuss below. This results in the default $\alpha_{\text{Max}} = 4 \times 10^5$.

2 Carrying capacity parameters

2.1 Using MRR data

There are four villages where population sizes have been estimated on multiple occasions (table 1). We estimated the parameters $\alpha_0, \alpha_1, \alpha_2, \phi, \kappa$ and δ using an MCMC algorithm as follows.

1. For each set of MRR population size estimates at a similar time of year, fit a Log-normal curve for the PDF of the 'true' population size for the given village and time of year (fig. 2). For the village Fourda (Baber et al., 2010), there was a single population size estimate for each of the dry and wet seasons, and we imposed a variance onto the estimated population sizes (in the other cases we used the sample variance from the different estimates across years).

2. Select a sample parameter set of $\{\alpha_0, \alpha_1, \alpha_2, \phi, \kappa, \delta\}$ (and default demographic parameters).

3. Run a simulation of the model using these parameters, over a simulation space that includes the four MRR villages. We let the simulation run for two years (the first 'warm-up' year is discarded).

4. Compute the 'likelihood' by comparing the simulated population sizes at the given dates to the PDFs from the data (fig. 2). Combine this with a prior distribution (fig. 3; these are more-or less uninformative except putting effective limits on the α's).

5. Repeat steps 2-4 iteratively, using a Metropolis-Hastings MCMC algorithm to estimate a posterior distribution.

This procedure was repeated with three simulation spaces:

1. Only the four focal villages included.

2. All settlements within 10km of each of the focal villages are included (102 settlements in total).

3. As 2, but a 20km radius of inclusion (429 settlements).

For the two larger simulation spaces, we set the dispersal parameter d at a high level of 0.01, which should emphasize the possible influence of neighbouring villages on each focal village.

2.1.1 Results

The maximum (pseudo) likelihood estimates for the six carrying capacity parameters are given in table 2. The parameters α_0 and δ showed little signs of converging in the MCMC experiments, reflecting that a.) a baseline level of breeding habitat is not necessary to fit the model to the focal populations, and b.) the relatively low level of non-permanent standing water (across the four villages) was not helpful to fitting the model.

The model fits best when allowing most of the variation in population sizes to be controlled purely by variation in rainfall, with a minor role given to permanent standing water. The optimal parameters are in the range of

$$\begin{aligned} \alpha_1 &= 300 \times 10^3 \\ \alpha_2 &= 50 \times 10^3 \\ \phi &= 0.02 \\ \kappa &= 0.5 \end{aligned}$$

It should be noted, however, that there is a strong covariance in the posterior between these parameter fits (for obvious reasons). A larger α_1 can be compensated by a smaller ϕ and similarly with α_2 and κ (though the signal is weaker; fig. 4).

2.2 Using intuition on population size distribution

It is likely that the MCMC algorithm permits a bias in the parameter estimates due to an over-reliance on a small number of data points which may themselves be inaccurate. For example, the algorithm predicts a low influence of water bodies due to the village with no water-bodies (Goundri) having high population estimates and the village with the most standing water (Fourda) having relatively small population estimates. Factors not accounted for may be partly responsible for this discrepancy (for example village size). It seems sensible to use the MCMC results as a guide rather than the final parameter choices.

Date	Season	Estimate	Reference	Model fit
**Banambini, Mali. Female MRR**				
July/August 1993	Wet	20×10^3	Touré et al. (1998)	
July/August 1994	Wet	64×10^3	Touré et al. (1998)	
July/August 1996	Wet	63×10^3	Touré et al. (1998)	86×10^3
July/August 1997	Wet	53×10^3	Taylor et al. (2001)	
July/August 1998	Wet	79×10^3	Taylor et al. (2001)	
**Goundri, Burkina Faso. Female MRR**				
September 1991	Wet	135×10^3	Costantini et al. (1996)	112×10^3
September 1992	Wet	330×10^3	Costantini et al. (1996)	
**Fourda, Mali. Female MRR**				
March 2008	Dry	5×10^3	Baber et al. (2010)	24×10^3
July 2009	Wet	30×10^3	Baber et al. (2010)	50×10^3
**Bana, Burkina Faso. Male MRR**				
May 2014	Dry	26×10^3	Epopa et al. (2017)	45×10^3
April 2015	Dry	194×10^3	Epopa et al. (2017)	
October 2013	Wet	158×10^3	Epopa et al. (2017)	172×10^3
September 2014	Wet	309×10^3	Epopa et al. (2017)	

Table 1: Population size estimates from MRR experiments and model fit values from the simulation model with default parameters. The model fit values are the mean of 10 replicate simulations that included all settlements within a 10km radius of the focal villages

The manipulate function (fig. 5) helps to explore the affect of each parameter on dry and wet season on population sizes across the simulation area. Playing with the parameters around the 'maximum-likelihood' suggests the following values give a sensible spread of population sizes:

$$\begin{aligned}
\alpha_0 &= 0 \\
\alpha_1 &= 2 \times 10^5 \\
\alpha_2 &= 2 \times 10^5 \\
\phi &= 0.03 \\
\kappa &= 0.8 \\
\delta &= 0.03.
\end{aligned}$$

In comparison with the MCMC results these parameters permit a larger influence of standing water on mosquito populations, yet rainfall *per se* remains the most important source of larval breeding habitat (fig. 5). Although we are setting $\alpha_0 = 0$ here, we will explore allowing some residual breeding habitat ($\alpha_0 > 0$) as a means to persist through the dry season.

3 Spatial parameters

3.1 d, probability adult disperses to a connected village

The role of this parameter will be explored across a wide range in the simulations, to reflect the great uncertainty in mosquito local dispersal.

3.2 L_d, maximum distance at which populations are connected to each other

The connections between local villages are weighted by distance ("Chinese-hat kernel"). We set $L_d = 10km$ on the basis that mosquitoes are unlikely to disperse further than this on a regular basis (other than by long-distance migration, which is treated separately). These assumptions give rise the distribution of migration distances shown in Additional file 2.

3.3 L_w, maximum distance at which populations are connected to water bodies

For each settlement, the perimeter length of standing water (both permanent and non-permanent) within the radius L_w is computed. We set $L_w = 5km$ on the basis that standing water beyond 5km is unlikely to have a major influence on the day to day dynamics of a population (other than by an indirect influence on neighbouring villages that are connected by dispersal).

4 Aestivation parameters

4.1 Aestivation timing $t_{A_1}, t_{A_2}, t_{A_3}, t_{A_4}$

Although the timing of the seasons varies to some extent across the simulation space, the timing of the nadir and peak of wetness is fairly consistent with the driest time $\approx$ week $1-10$ and wettest $\approx$ week $35-40$ (fig. 6). We set the aestivation period to begin at the end of the wet season on 27th October and end before the nadir of the wet season on 16th December. Adult females emerge from aestivation early in the wet season, between 20th May and the 19th June.

4.2 Probability of entering and surviving aestivation ψ and μ_E

We set $\psi = 0$ (no aestivation) as a default but will explore a wide range of this parameter. Similarly we explore a wide range of aestivation mortality.

5 Long-distance migration parameters

Surface winds in the Sahel follow a yearly fluctuation (Nicholson, 2013), and it has been hypothesised that this is used by mosquitoes for an annual two-way migration. We use the yearly wind pattern described by Nicholson (2013) to set the timing of this return trip, giving $t_{D_1} = 1^{st}$ January, $t_{D_2} = 30^{th}$ January, $t_{D_3} = 20^{th}$ July, and $t_{D_4} = 19^{th}$ August.

As with aestivation, we set a default of no migration ($d_M = 0$) yet explore a wide range of migration propensity, and also explore a wide range of migration survival. The distribution of migration distances (in either direction) in a simulation run are shown in Additional file 2.

References

Baber, I., M. Keita, N. Sogoba, M. Konate, S. Doumbia, S. F. Traoré, J. M. Ribeiro, N. C. Manoukis, et al., 2010. Population size and migration of anopheles gambiae in the bancoumana region of mali and their significance for efficient vector control. PLoS One 5:e10270.

Costantini, C., S.-G. LI, A. D. Torre, N. Sagnon, M. Coluzzi, and C. E. Taylor, 1996. Density, survival and dispersal of anopheles gambiae complex mosquitoes in a west african sudan savanna village. Medical and veterinary entomology 10:203–219.

Epopa, P. S., A. A. Millogo, C. M. Collins, A. North, F. Tripet, M. Q. Benedict, and A. Diabate, 2017. The use of sequential mark-release-recapture experiments to estimate population size, survival and dispersal of male mosquitoes of the anopheles gambiae complex in bana, a west african humid savannah village. Parasites & vectors 10:376.

Gary, R. E. and W. A. Foster, 2001. Effects of available sugar on the reproductive fitness and vectorial capacity of the malaria vector anopheles gambiae (diptera: Culicidae). Journal of Medical Entomology 38:22–28.

Gimnig, J. E., M. Ombok, S. Otieno, M. G. Kaufman, J. M. Vulule, and E. D. Walker, 2002. Density-dependent development of anopheles gambiae (diptera: Culicidae) larvae in artificial habitats. Journal of Medical Entomology 39:162–172.

Manda, H., L. C. Gouagna, W. A. Foster, R. R. Jackson, J. C. Beier, J. I. Githure, and A. Hassanali, 2007. Effect of discriminative plant-sugar feeding on the survival and fecundity of anopheles gambiae. Malaria journal 6:113.

Muriu, S. M., T. Coulson, C. M. Mbogo, and H. C. J. Godfray, 2013. Larval density dependence in anopheles gambiae ss, the major african vector of malaria. Journal of Animal Ecology 82:166–174.

Nicholson, S. E., 2013. The west african sahel: A review of recent studies on the rainfall regime and its interannual variability. ISRN Meteorology 2013.

Taylor, C., Y. T. Touré, J. Carnahan, D. E. Norris, G. Dolo, S. F. Traoré, F. E. Edillo, and G. C. Lanzaro, 2001. Gene flow among populations of the malaria vector, anopheles gambiae, in mali, west africa. Genetics 157:743–750.

Touré, Y. T., G. Dolo, V. Petrarca, A. Dao, J. Carnahan, and C. E. Taylor, 1998. Mark-release-recapture experiments with anopheles gambiae sl in banambani village, mali, to determine population size and structure. Medical and veterinary entomology 12:74–83.

Yaro, A., A. Dao, A. Adamou, J. Crawford, S. Traore, A. Toure, R. Gwadz, and T. Lehmann, 2006. Reproductive output of female anopheles gambiae (diptera: Culicidae): comparison of molecular forms. Journal of medical entomology 43:833–839.

Yaro, A. S., A. I. Traoré, D. L. Huestis, A. Adamou, S. Timbiné, Y. Kassogué, M. Diallo, A. Dao, S. F. Traoré, and T. Lehmann, 2012. Dry season reproductive depression of anopheles gambiae in the sahel. Journal of insect physiology 58:1050–1059.

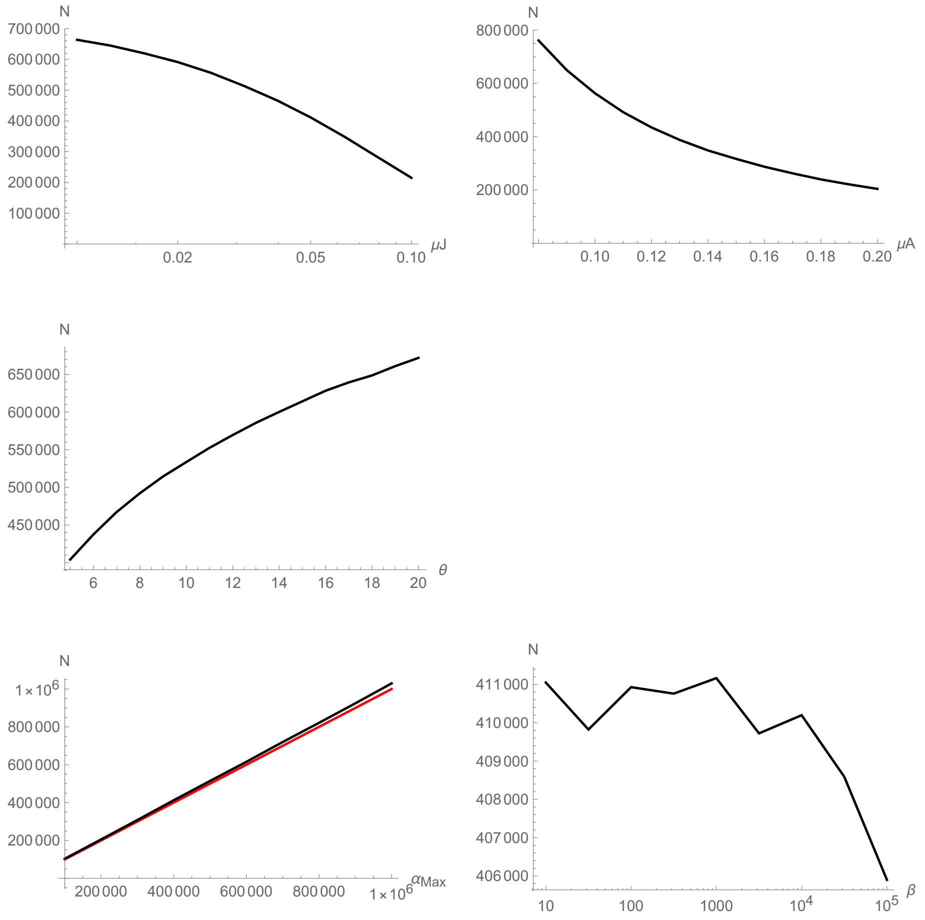

Figure 1: Effects of varying the demographic parameters on population size (number of adult females in a single population) when all the others are at baseline levels. Note that these are population sizes that are attained in the most favourable possible conditions (normally $\alpha(x,t) < \alpha_{\mathrm{Max}}$). For these parameters, we find population size $\approx \alpha_{\mathrm{Max}}$ (red line shows $y = x$).

Parameter	Focal villages only 5000 iterations	Focal +10km radius 5000 iterations	Focal +20km radius 500 iterations
α_0	128	2	16
α_1	291×10^3	294×10^3	334×10^3
α_2	38.1×10^3	52.2×10^3	35.2×10^3
δ	0.190	0.0189	0.221
ϕ	0.0225	0.0218	0.0187
κ	0.503	0.401	0.503

Table 2: Maximum likelihood estimates for carrying capacity parameters from MCMC algorithm using MRR population size estimates

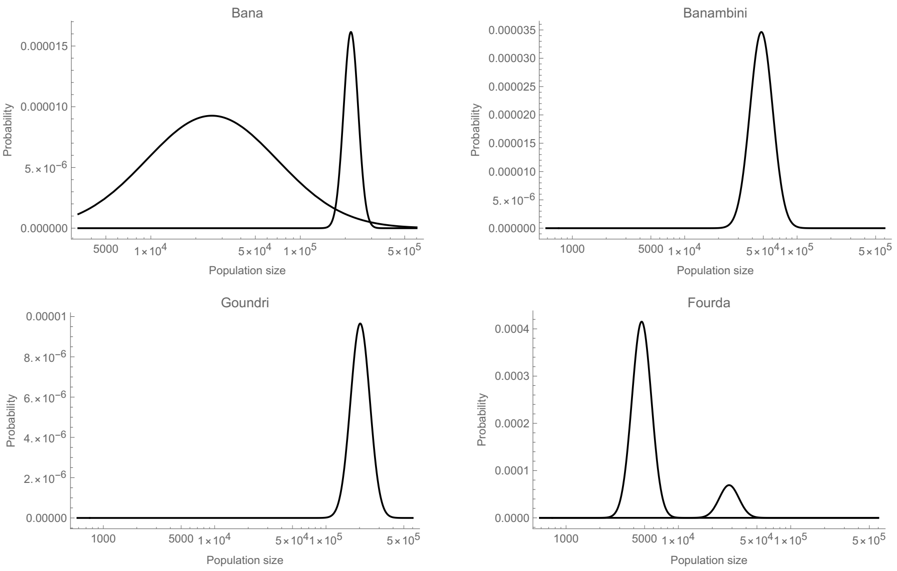

Figure 2: Probability density functions for population sizes in four villages, derived from MRR data. The two curves for Bana and Fourda relate dry and wet season populations, while the Goundri and Banambini curves are wet season populations (see table 1).

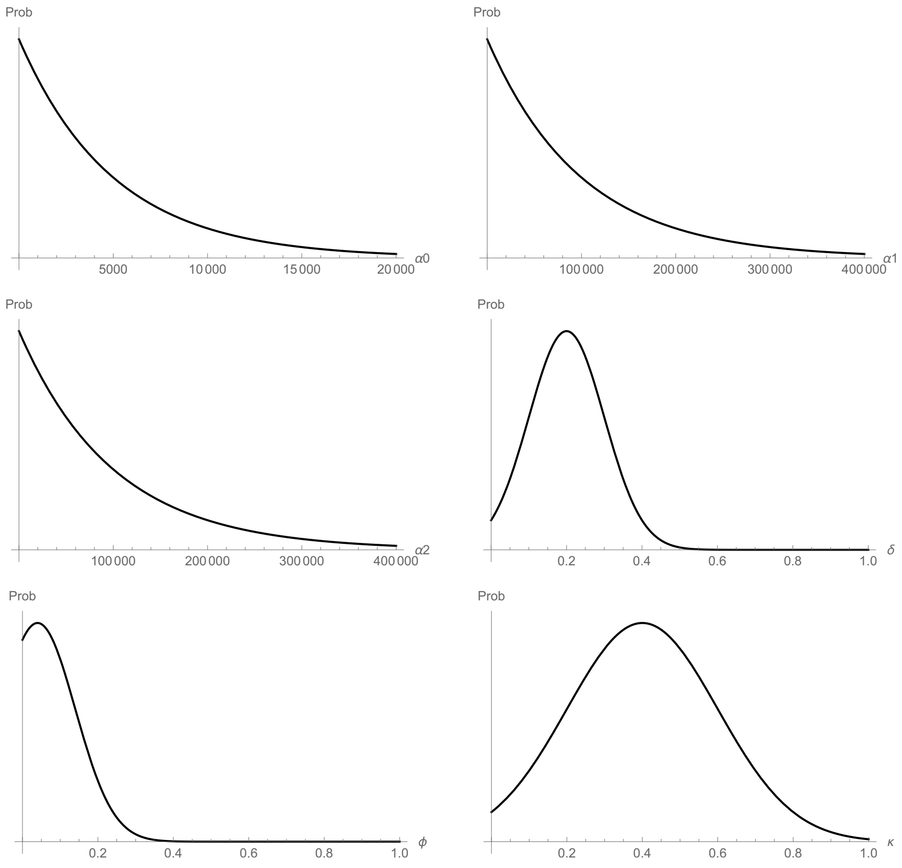

Figure 3: Prior distributions for the carrying capacity parameters

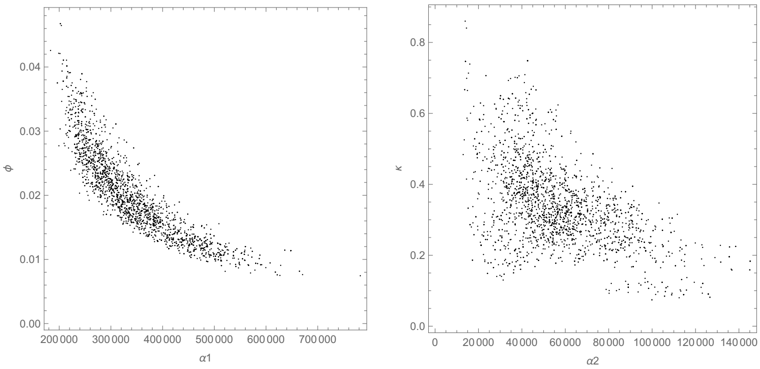

Figure 4: Posterior distribution from the 10km MCMC experiment

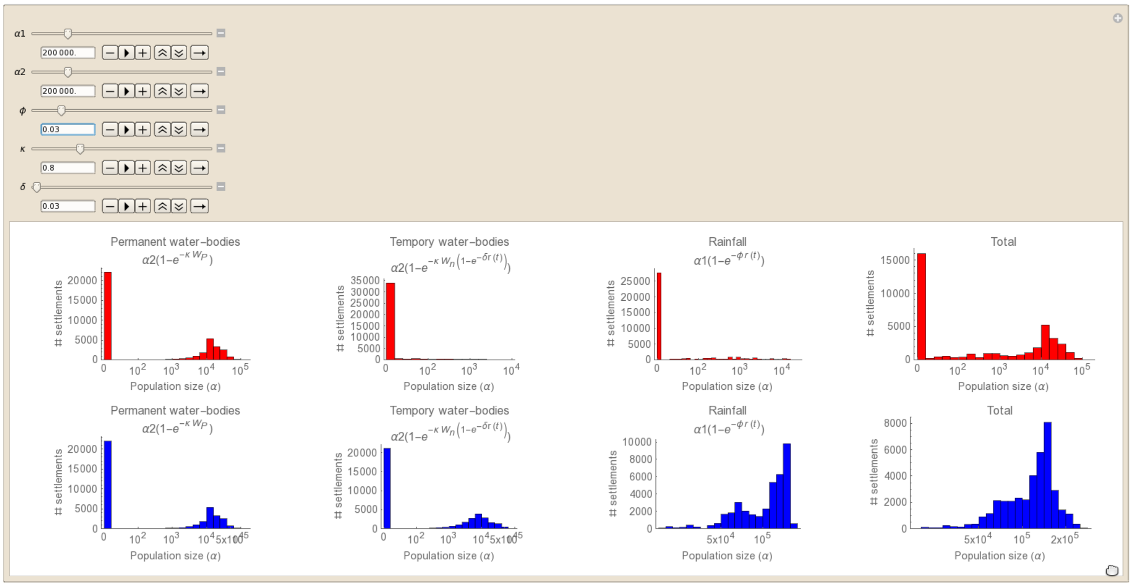

Figure 5: Manipulate function to explore contributions to adult female population size from each component. Red is dry season and blue is wet season. The x-axis plots $\alpha(t)$, yet this is a good proxy for adult female population size - compare red and black lines in fig. 1

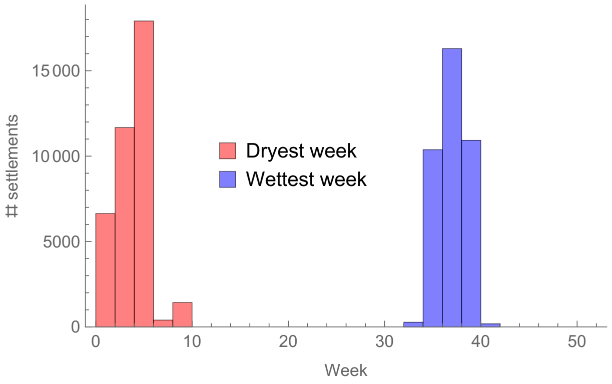

Figure 6: Timing of wettest and driest week across settlements
