# Supplementary material for: Modelling the persistence of mosquito vectors of malaria in Burkina Faso
Source: Malar J. 2018 Apr 2;17:140. doi: 10.1186/s12936-018-2288-3 (PMC5879775; doi:10.1186/s12936-018-2288-3)
Supplement: Supplementary file 5 — Additional file 5. The effects of combining dry season survival hypotheses (aestivation, migration and small permanent larval sites) on vector persistence across the simulation area. [file 12936_2018_2288_MOESM5_ESM.pdf]

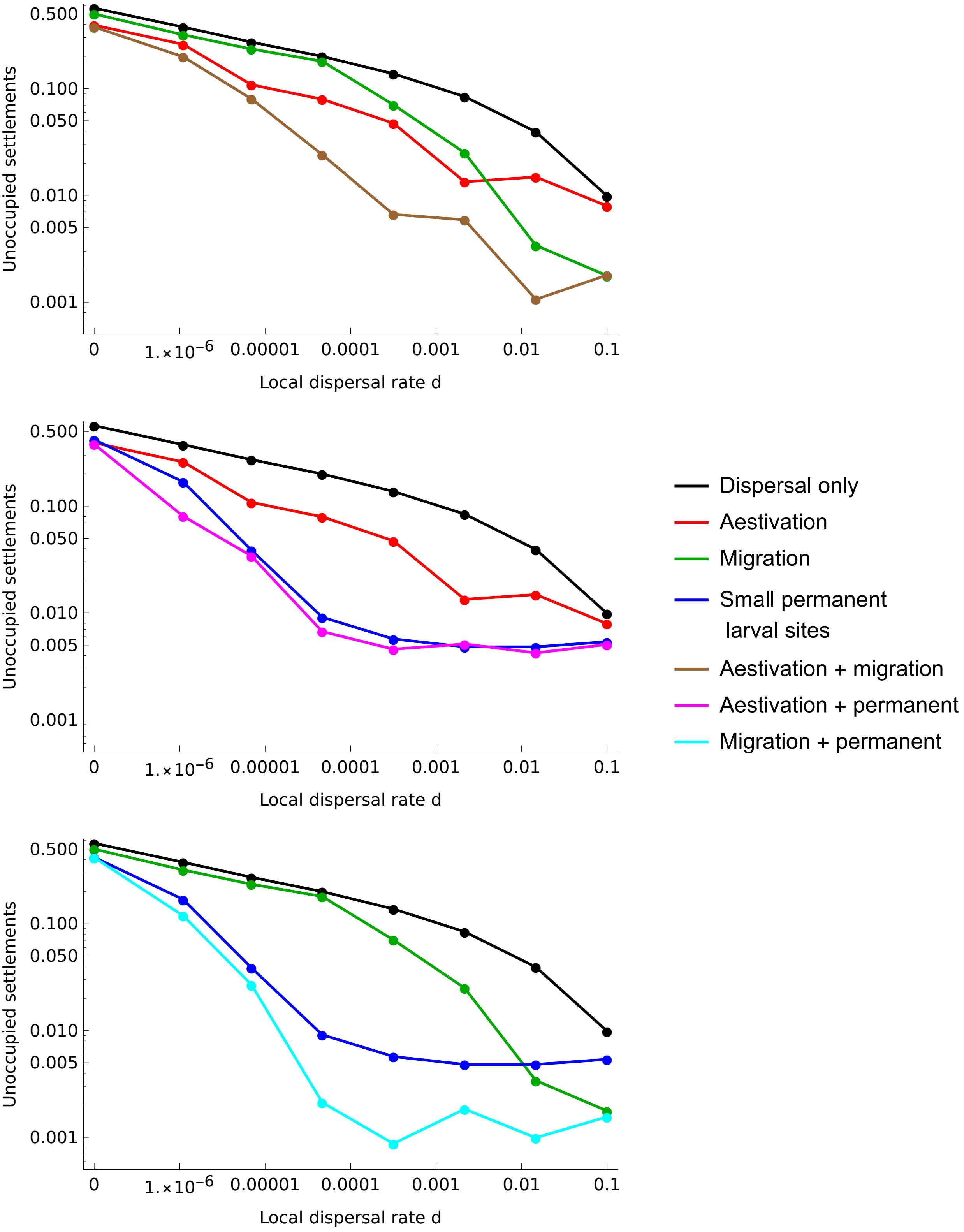

Figure S3: The effect of combining hypotheses to explain vector persistence, over a range of local dispersal frequencies. Aestivation parameters are $\psi = 10^{-3}, \mu_E = 0.9$, migration parameters are $d_L = 10^{-5}, \mu_M = 0.99$, and residual water parameters are $\alpha_0^{\mu} = 4, \alpha_0^{\sigma^2} = 10$.
